# Circulating tumor DNA and magnetic resonance imaging to predict neoadjuvant chemotherapy response and recurrence risk

**DOI:** 10.1038/s41523-021-00239-3

**Published:** 2021-03-25

**Authors:** Mark Jesus M. Magbanua, Wen Li, Denise M. Wolf, Christina Yau, Gillian L. Hirst, Lamorna Brown Swigart, David C. Newitt, Jessica Gibbs, Amy L. Delson, Ekaterina Kalashnikova, Alexey Aleshin, Bernhard Zimmermann, A. Jo Chien, Debu Tripathy, Laura Esserman, Nola Hylton, Laura van ‘t Veer

**Affiliations:** 1 Department of Laboratory Medicine, University of California San Francisco, San Francisco, CA USA; 2 Department of Radiology and Biomedical Imaging, University of California San Francisco, San Francisco, CA USA; 3 Department of Surgery, University of California San Francisco, San Francisco, CA USA; 4 Breast Science Advocacy Core, University of California San Francisco, San Francisco, CA USA; 5 Natera Inc., San Ramon, CA USA; 6 Division of Hematology Oncology, University of California San Francisco, San Francisco, CA USA; 7 Department of Breast Medical Oncology, The University of Texas MD Anderson Cancer Center, Houston, TX USA

**Keywords:** Tumour biomarkers, Prognostic markers, Predictive markers, Cancer imaging

## Abstract

We investigated whether serial measurements of circulating tumor DNA (ctDNA) and functional tumor volume (FTV) by magnetic resonance imaging (MRI) can be combined to improve prediction of pathologic complete response (pCR) and estimation of recurrence risk in early breast cancer patients treated with neoadjuvant chemotherapy (NAC). We examined correlations between ctDNA and FTV, evaluated the additive value of ctDNA to FTV-based predictors of pCR using area under the curve (AUC) analysis, and analyzed the impact of FTV and ctDNA on distant recurrence-free survival (DRFS) using Cox regressions. The levels of ctDNA (mean tumor molecules/mL plasma) were significantly correlated with FTV at all time points (*p* < 0.05). Median FTV in ctDNA-positive patients was significantly higher compared to those who were ctDNA-negative (*p* < 0.05). FTV and ctDNA trajectories in individual patients showed a general decrease during NAC. Exploratory analysis showed that adding ctDNA information early during treatment to FTV-based predictors resulted in numerical but not statistically significant improvements in performance for pCR prediction (e.g., AUC 0.59 vs. 0.69, *p* = 0.25). In contrast, ctDNA-positivity after NAC provided significant additive value to FTV in identifying patients with increased risk of metastatic recurrence and death (*p* = 0.004). In this pilot study, we demonstrate that ctDNA and FTV were correlated measures of tumor burden. Our preliminary findings based on a limited cohort suggest that ctDNA at surgery improves FTV as a predictor of metastatic recurrence and death. Validation in larger studies is warranted.

## Introduction

Neoadjuvant chemotherapy (NAC) has become a standard-of-care for early breast cancer patients diagnosed with locally advanced disease^1^. Since NAC is administered prior to surgical resection of the primary tumor, it offers a unique window for real-time monitoring of tumor response during treatment^2–4^. Approximately 10–65% of patients—depending on subtype and treatment—achieve pathologic complete response (pCR) after NAC^5^. pCR is characterized by the complete eradication of invasive cancer in the breast and regional nodes. A pooled analysis by Spring and colleagues has shown that achieving pCR provides a significant survival advantage^6^. More recent results from the I-SPY 2 TRIAL (NCT01042379), a multicenter phase 2 trial that evaluates standard NAC in combination with investigational therapies, have shown that patients who achieve pCR have more favorable outcomes (95% distant recurrence-free survival or DRFS) at 3 years compared to non-responders^7^.

Thus, a major challenge faced by clinicians in the neoadjuvant setting is how to enable each patient to achieve pCR while minimizing exposure to treatment-related toxicities. Biomarkers that accurately predict response to NAC early during treatment are key to personalizing therapy, as non-responders could be eligible for an early switch to a more effective therapy to increase the likelihood of achieving a pCR and responders could potentially be sent to surgery early (de-escalation). Furthermore, additional chemotherapy and targeted therapies in the post-neoadjuvant setting have been shown to improve long term outcomes of non-responders^8^. For patients who ultimately do not achieve a pCR, discovering biomarkers that can identify those at highest risk of relapse are unmet clinical needs.

Compared to clinical exam, mammography, and ultrasound, magnetic resonance imaging (MRI) is the most accurate imaging tool in monitoring tumor response to treatment in NAC^9^. Previous studies showed that MRI-based functional tumor volume (FTV) can predict pCR and recurrence-free survival for patients with invasive breast cancer undergoing NAC^10–12^. In the I-SPY 2 TRIAL, serial MRI exams has been implemented to monitor treatment response and patient randomization^13^.

In addition to imaging, the I-SPY 2 TRIAL has actively examined molecular biomarkers as predictors of response and recurrence^14,15^, including circulating tumor DNA (ctDNA)^16^. Clinical studies have shown that ctDNA analysis in blood offers a promising minimally invasive approach for real-time disease monitoring and evaluation of response to treatment^17,18^. For example, in I-SPY 2, we recently reported on a serial analysis of ctDNA and demonstrated that among patients who were ctDNA-positive at baseline, those who cleared ctDNA early during treatment (3 weeks after initiation of treatment) were more likely to achieve pCR than patients who remained ctDNA-positive^16^. We also showed that residual ctDNA after NAC but before surgery was strongly associated with poor DRFS^16^. Other ctDNA studies in the neoadjuvant setting in breast cancer have revealed that ctDNA-positivity before NAC was associated with a decreased likelihood of achieving a pCR and increased ctDNA levels after NAC was correlated with poor response^19–21^.

While previous studies have shown that FTV^10,11^ and ctDNA^16^ are independently associated with response to NAC and survival, the added value of combining both measures to improve prediction of response and outcome has yet to be fully explored.

We performed a pilot study in a subset of patients in the I-SPY 2 TRIAL to (1) examine the correlation between ctDNA and FTV at different time points during NAC (2) investigate whether FTV and ctDNA dynamics are correlated; and (3) determine whether combining information from these two measurements can improve prediction of response to NAC and estimation of risk of recurrence.

We hypothesized that a multimodal approach for monitoring tumor response during NAC—i.e., by FTV and ctDNA analyses—can yield robust and accurate predictors of response to NAC and DRFS, and ultimately aid in therapeutic decisions regarding modification or de-escalation of therapy (treatment redirection) to improve patient outcomes.

## Results

### Patient and tumor characteristics

The mean age of the 84 patients in the study was 49 (range: 25–73 years old). Of the 84, 35% had hormone receptor-positive/HER2-negative, 23% HER2-positive, and 43% triple negative breast cancers; 61% of tumors were MammaPrint high 2 (ultra-high risk), 30% stage T3 or T4, and 53% node-negative (Table 1).

**Table 1:** Patient and tumor characteristics.

Characteristic	*N* = 84
Mean age (range)	49.3 (25–73)
	*N* (%)
Treatment	
Standard NAC	32 (38%)
Standard NAC + MK-2206	52 (62%)
Subtype	
HR+HER2−	29 (35%)
HER2+	19 (23%)
TNBC	36 (43%)
Clinical T stage (n = 78)	
T1/T2	55 (71%)
T3/T4	23 (29%)
Clinical N stage (*n* = 74)	
Negative	39 (53%)
Positive	35 (47%)
Grade (*n* = 56)	
II	22 (39%)
III	34 (61%)
MammaPrint score	
High 1	33 (39%)
High 2	51 (61%)
Pathologic complete response (pCR)	
pCR	23 (27%)
no pCR	61 (73%)

*NAC* neoadjuvant chemotherapy, *HR+* hormone receptor-positive, *TNBC* triple negative breast cancer. Percentages may not equal to 100 due to rounding.

### ctDNA, FTV and clinicopathologic variables

**Fig. 1:**
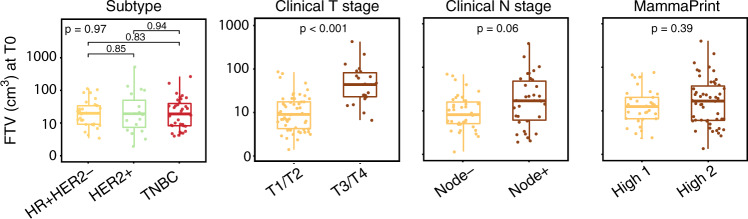
Pretreatment functional tumor volume (FTV) and association with clinicopathologic characteristics. *P* values were calculated using Mann–Whitney (2 groups) or Kruskal–Wallis test (3 groups). For each box plot, the center line, the boundaries of the box, the ends of the whiskers and points beyond the whiskers represent the median value, the interquartile range, the minimum and maximum values, and the outliers, respectively.

We have previously shown that pretreatment ctDNA (as binary or continuous variable) was associated with subtype, clinical T stage, and MammaPrint score (as risk categories MammaPrint high 1 and high 2)^16^. Here, we examined associations between pretreatment FTV and clinicopathologic variables. As expected, we found that FTV was significantly associated with clinical T stage (*p* < 0.001) (Fig. 1). FTV was not significantly associated with subtype (*p* = 0.97), clinical N stage (*p* = 0.06), and MammaPrint score (*p* = 0.39).

### ctDNA and FTV are correlated measures of tumor burden

**Fig. 2:**
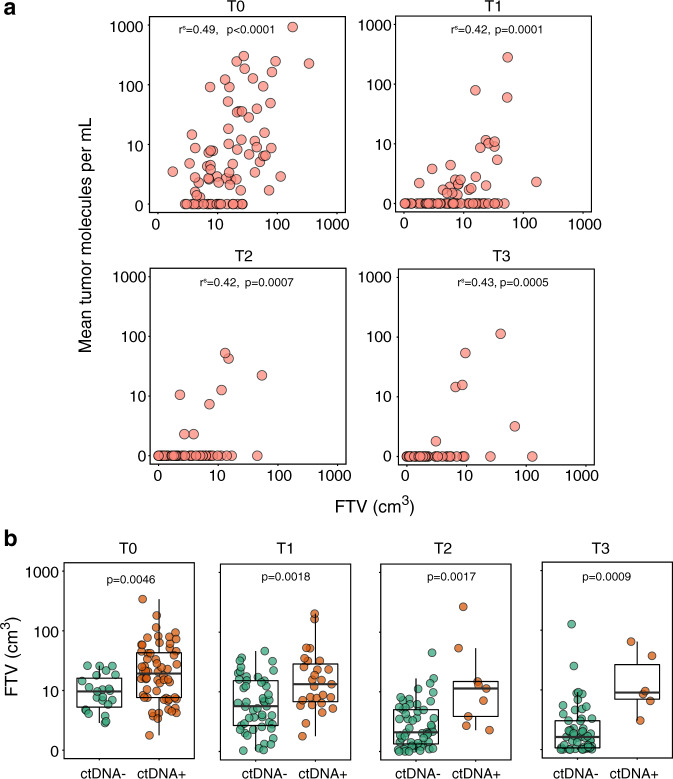
Relationship between ctDNA and FTV at different time points. **a** Scatterplot for all patients with paired data at different time points to show correlation between ctDNA and MRI-based FTV as measures of tumor burden. **b** ctDNA-positivity and tumor burden by MRI-based FTV. Box plots showing distribution of FTV according to ctDNA status (ctDNA-positive or ctDNA-negative) at different time points. T0: pretreatment; T1: 3 weeks after initiation of paclitaxel-based treatment; T2: between paclitaxel and anthracycline regimens; T3: after NAC prior to surgery. For each box plot, the center line, the boundaries of the box, the ends of the whiskers and points beyond the whiskers represent the median value, the interquartile range, the minimum and maximum values, and the outliers, respectively.

We examined whether ctDNA and FTV measurements were correlated at different time points during NAC. Scatter plots in Fig. 2A show the relationship between FTV (cm^3^) and ctDNA (MTM/mL) at each time point. We found that the ctDNA concentration was significantly correlated with FTV at all time points [T0 (ρ = 0.49, *p* < 0.0001), T1 (ρ = 0.42, *p* = 0.0001), T2 (ρ = 0.42, *p* = 0.0007), T3 (ρ = 0.43, *p* = 0.0005)].

We also examined whether ctDNA-positivity at each time point was associated with higher tumor burden as measured by FTV. The median FTV among patients who had detectable ctDNA (ctDNA-positive) was significantly higher compared to the median FTV of those who were ctDNA-negative at all time points: [T0 (*p* = 0.0046), T1 (*p* = 0.0018), T2 (*p* = 0.0017), T3 (*p* = 0.0009] (Fig. 2B).

### Correlation between ctDNA and FTV dynamics during NAC

**Fig. 3:**
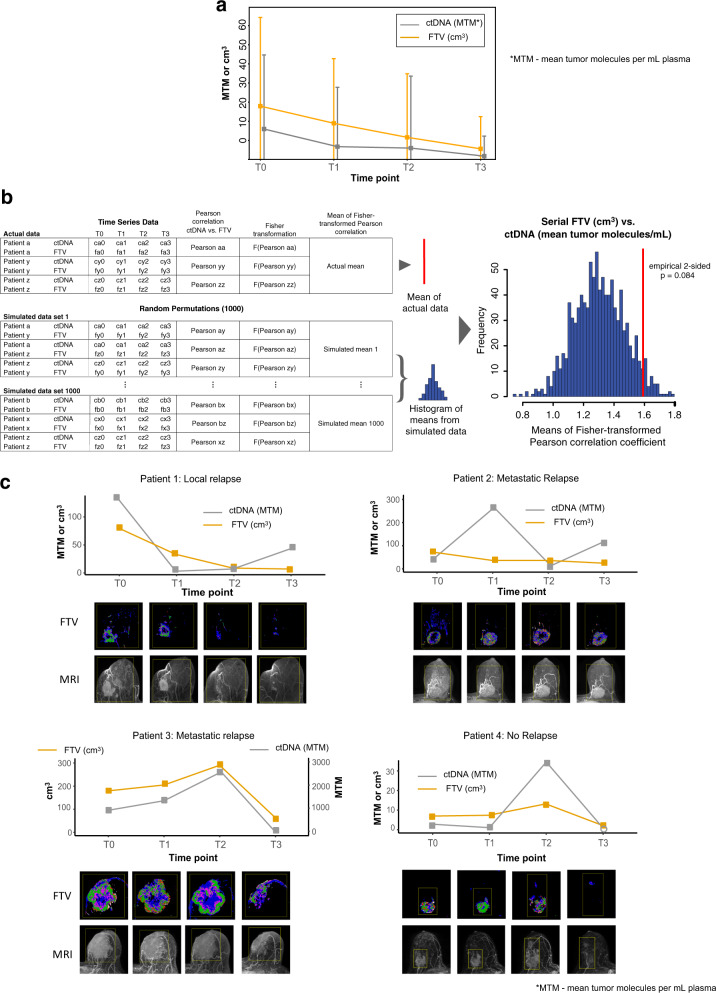
Correlation between ctDNA and MRI-based FTV dynamics during NAC. **a** Mean levels of ctDNA (mean tumor molecules per mL plasma, MTM) and FTV (cm^3^) across time points. The error bars represent standard deviation. The vertical lines represent standard deviation. **b** Schematic for calculating the mean Pearson correlation for actual data and means for simulated datasets using Monte Carlo method. The right panel is histogram showing the distribution of simulated means of Fisher z-transformed Pearson’s correlation coefficient. The red line corresponds to the mean Fisher z-transformed Pearson’s correlation coefficient calculated based on the actual data. **c** Plots showing ctDNA (mean tumor molecules per mL plasma, MTM) and FTV (cm^3^) levels across time points in 4 representative cases. Unfilled dot at T3 in patient 4 indicates ctDNA below the limit of detection. T0: pretreatment; T1: 3 weeks after initiation of paclitaxel-based treatment; T2: between paclitaxel and anthracycline regimens; T3: after NAC prior to surgery.

We investigated whether ctDNA and FTV dynamics (trajectory patterns during treatment) were correlated. Overall, the mean values of ctDNA and FTV both decreased during treatment (Fig. 3A). To determine the correlation between ctDNA and FTV trajectories at the individual patient level, we performed Monte Carlo simulation to calculate an empirical *p* value, as described in the Methods. Figure 3B shows the mean Fisher z-transformed Pearson correlation coefficient between ctDNA and FTV in the actual population (red line, mean = 1.59) relative to its distribution in the simulated data set (histogram). We found that both FTV and ctDNA trajectories in individual patients showed a general decrease over time. However, the correlation between these two variables did not reach statistical significance (empirical two-sided *p* = 0.084).

Examples of cases with complete serial ctDNA and FTV data are shown in Fig. 3C. Overall, FTV and ctDNA dynamics showed similarities for each of these patients.

*Patient 1* showed a steady decrease in FTV across time points, while her ctDNA levels substantially dropped early in treatment (T1), and then increased as treatment progressed. The patient experienced local recurrence one year and two months after study entry.

FTV in *Patient 2* decreased during NAC, while ctDNA trajectory was less consistent. An early spike in ctDNA and subsequent decrease after paclitaxel-based treatment were observed. Increase in ctDNA levels was observed after anthracycline treatment. The patient eventually experienced a metastatic relapse a year after study entry.

In *Patient 3*, the FTV and ctDNA followed the same dynamics; both increased during paclitaxel-based treatment and decreased after anthracycline treatment. ctDNA levels (in absolute value) were higher by at least an order of magnitude compared to FTV. While there was a downward trend of both ctDNA and FTV toward the end of NAC, the patient still had detectable ctDNA and was deceased 2 months after surgery.

*Patient 4* experienced a dramatic spike in ctDNA levels after paclitaxel-based treatment. A similar pattern was observed for FTV. The patient eventually cleared ctDNA after NAC (T3). No relapse was documented for this patient as of the last follow-up (3 years and 6 months after study entry).

### Additive value of ctDNA to FTV for prediction of response to NAC

**Fig. 4:**
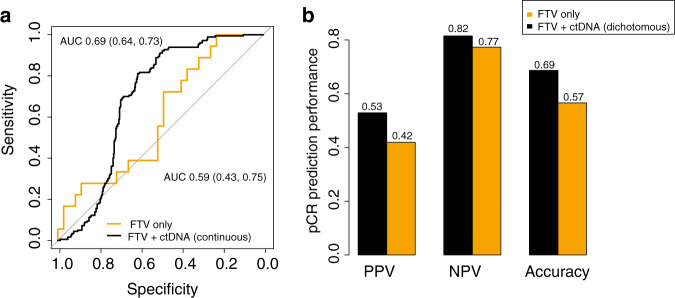
Additive value of ctDNA to FTV-based predictors of pCR at T1. **a** Area under the curve (AUC) for prediction of pCR early during NAC (T1: 3 weeks after initiation treatment) for FTV alone and FTV + ctDNA treated as a continuous variable. **b** Positive predictive value (PPV), negative predictive value (NPV) and accuracy for prediction of pCR of FTV alone and FTV + ctDNA treated as a dichotomous variable.

We evaluated whether ctDNA information could improve the performance of FTV-based predictors for pCR prediction. Of the 53 patients who were ctDNA-positive at baseline, 18 (34%) achieved a pCR. Adding the ctDNA variables baseline value and percent change between T0 and T1, to the FTV-based predictors improved the T1 AUC (FTV: 0.59, FTV + ctDNA: 0.69, *p* = 0.25) in this subset, but the change was not statistically significant (Fig. 4A, Supplementary Table 1). Optimal dichotomization of this FTV + ctDNA combined model showed numerical improvements in predictive performance (positive predictive value, negative predictive value, and accuracy) relative to the FTV-only model as well (Fig. 4B). No improvements in prediction performance were observed at other time points (Supplementary Table 1).

### Additive value of ctDNA to FTV as a prognostic factor of survival

DRFS data were available for 75 of the 84 patients: 10 experienced distant metastases, of whom 8 died. The median follow-up was 4.8 years. Of the 75, 70 had measurable FTV at T3.

We assessed the prognostic significance of ctDNA and FTV measured at T3 (after NAC) and standard clinicopathologic variables using Cox regressions. Univariable analysis showed that FTV (Hazard ratio: 1.03, 95% CI: 1.01–1.04, *p* = 0.0005) and ctDNA (Hazard ratio: 11.50, 95% CI: 2.87–46.14, *p* = 0.0006) were significantly associated with DRFS (Supplementary Table 2). Bivariable analysis showed significant association between FTV (Hazard ratio: 1.03, 95% CI: 1.01–1.05, *p* = 0.0005) and ctDNA status at T3 (Hazard ratio: 13.40, 95% CI: 2.97–60.50, *p* = 0.0007) and DRFS. There was no significant interaction between FTV and ctDNA (*p* = 0.1245, Table 2).

**Table 2:** Bivariate analysis to test for main effects and interaction between circulating tumor DNA (ctDNA) and functional tumor volume (FTV) by magnetic resonance imaging.

Bivariate analysis at T3 (*n* = 58, number of events = 8)	Without interaction term				With interaction term			
	Hazard ratio	Lower 95% CI	Upper 95% CI	Wald test *p* value	Hazard ratio	Lower 95% CI	Upper 95% CI	Wald test *p* value
FTV (continuous)	1.03	1.01	1.05	0.0005	1.03	1.01	1.05	0.0063
ctDNA (dichotomous)	13.40	2.97	60.50	0.0007	4.89	0.64	37.17	0.1252
FTV * ctDNA	−	−	−	−	1.07	0.98	1.16	0.1245

T3 represents the time point after neoadjuvant chemotherapy prior to surgery.

*CI* confindence interval

In an exploratory multivariable analysis, FTV (Hazard ratio: 1.03, 95% CI: 1.01–1.10, *p* = 0.0191) and ctDNA (Hazard ratio: 14.25, 95% CI: 2.27–89.3, *p* = 0.0046) remained significant predictors of DRFS (Supplementary Table 2). Addition of ctDNA to a Cox model that included pCR, subtype and FTV resulted in the lowest AIC score (AIC = 55.85, *p* = 0.004), indicating best fit to the survival data, and therefore could best predict DRFS (Supplementary Table 3).

## Discussion

This study aimed to develop strategies for combining imaging (FTV by MRI) and liquid biopsy (ctDNA) data to build predictive models of response to NAC and survival. It was performed in the context of the I-SPY 2 TRIAL, a multicenter trial that evaluates therapeutic agents in the neoadjuvant setting.

Here, we report on studies involving serial measurements of ctDNA and FTV for monitoring tumor response in 84 high-risk early breast cancer patients who received NAC in the I-SPY 2 TRIAL. We demonstrate how ctDNA information may be used to improve the performance of FTV as a predictor of pCR and DRFS. This exploratory study provides a logistic and analytic framework for larger studies designed to develop minimally invasive approaches (i.e., tissue biopsy-free) that combine imaging and liquid biopsy biomarkers for early prediction of pCR and outcome.

In this study, we used two previously clinically validated measures of tumor burden: (1) FTV, an MRI-based measure of solid tumor burden, and (2) Signatera ctDNA test, a liquid biopsy-based measure of circulating tumor burden.

FTV is used in the adaptive randomization algorithm of the I-SPY 2 TRIAL under Food and Drug Administration (FDA) Investigation Device Exemption^2,22,23^. FTV is measured using standardized MR image acquisition, analysis, and interpretation. FTV, however, measures disease extent only at the macroscopic level. The accuracy for predicting pCR and survival varies among molecular subtypes, tumor morphology, and treatment time points^17,18^. In contrast to FTV, ctDNA analysis measures treatment response and residual disease after NAC at the molecular level, and thus can potentially complement FTV in the prediction of pCR or survival.

In 2019, the Signatera test was granted “Breakthrough Device” designation by the FDA and its clinical validity for post-surgical detection of residual disease (ctDNA) and relapse monitoring has been established across cancer types including breast cancer^24–27^. ctDNA testing predicted disease relapse significantly earlier than conventional imaging^25–27^. While most ctDNA technology platforms monitor a fixed panel of therapeutically relevant genes (e.g., TEC-seq^28^, Safe-Seq^29^, Guardant360^30^, CAPP-seq^31^), the Signatera test is unique as it involves a customized design of the assay (16-plex) to match the unique signature of clonal variants found in each individual’s tumor^24–27^.

Results from our study revealed that ctDNA and FTV were correlated measures of tumor burden. We observed numerical increases in AUC in pCR prediction models that include both ctDNA and FTV versus models with FTV alone; however, the improvements in AUCs were not statistically significant.

Studies involving 950 patients in the I-SPY 2 TRIAL have demonstrated that pCR and subtype are significant predictors of DRFS^7^. In this limited cohort, we did not observe the prognostic impact of these variables. Exploratory multivariable analysis showed that ctDNA and FTV after NAC remained significant prognostic factors for DRFS after adjusting for potential confounders.

Taken together, findings based on a limited number of DRFS events provided evidence for the additive value of ctDNA to FTV-based predictors for predicting recurrence. Preliminary studies did not show a significant additive value of ctDNA to FTV for prediction of pCR. This is likely because high FTV and ctDNA-positivity during treatment are both residual disease measures that associate with non-response.

While this study focused only on the demonstrated imaging metric FTV, additional metrics derivable from both DCE-MRI and diffusion-weighted MRI, currently being developed and tested, can be included as they become reliably measured and standardized in I-SPY 2^23,32^. Future studies will evolve with the development and validation of imaging biomarkers to leverage important breakthroughs in ctDNA research. Our ultimate goal is to develop a pCR prediction tool to identify good responders who may be eligible for early surgical treatment to reduce exposure to toxicities from unnecessary additional therapies; and poor responders who may benefit from a change in therapy to increase the likelihood of achieving a pCR.

The idea of a blood-based test that complements and reduces the need for repeated MRI to monitor disease burden and treatment response is very attractive. More frequent and intermediate monitoring of ctDNA can be performed between the sparse imaging assessments. While ctDNA and imaging can be used in tandem, we envisage that serial analysis of blood-based biomarkers (e.g., ctDNA) would be easier to perform and thus be more appealing to patients.

The study was limited by the modest sample size and few DRFS events. Confirmatory studies in larger cohorts to examine the clinical utility of combined ctDNA and FTV tests in different subtypes of breast cancers are warranted. ctDNA experiments are currently underway to expand the study to other arms of the I-SPY 2 TRIAL. With increased sample size and number of events, we can control for the effects of treatment and other clinicopathologic variables on our prognostic models.

Our pilot study features an innovative use of an existing, clinically validated imaging measure of tumor burden (i.e., MRI-based FTV) combined with an emerging liquid biopsy technology (Signatera, ctDNA test) to assess tumor response early during treatment and measure residual cancer after NAC. With DCE-MRI as the starting point, we ultimately aim to establish the combined strategy (imaging + liquid biopsy) as the enabling technologies for treatment redirection during NAC as well as guiding therapeutic decision-making in the adjuvant setting. Our approach capitalizes on the reliability of well-developed imaging methods to enable rapid implementation, while pursuing the use of a minimally invasive liquid biopsy-based modality for measurement of tumor burden.

## Methods

### Patient population

A total of 84 women with high-risk (stage II or III) early breast cancer from the I-SPY 2 TRIAL (NCT01042379) who had paired ctDNA and FTV data were included for this study. Patients in this cohort received paclitaxel alone (*n* = 32) or paclitaxel plus MK-2206 (*n* = 52), an AKT inhibitor, followed by anthracycline-based treatment^33^.

### Ethics declaration

All participating sites (University of California San Francisco, MD Anderson Cancer Center; Loyola University, University of California San Diego, University of Alabama at Birmingham, Swedish Cancer Institute, University of Chicago Medical Center, University of Colorado Denver, University of Texas Southwestern, Oregon Health & Science University, Georgetown University, University of Pennsylvania, University of Southern California, Cancer Therapy Evaluation Program, Inova Health System, Mayo Clinic, University of Arizona, Masonic Cancer Center, University of Minnesota) received approval from an institutional review board. All patients signed informed consent to allow research on and use of their biospecimen samples.

### Data acquisition

**Fig. 5:**
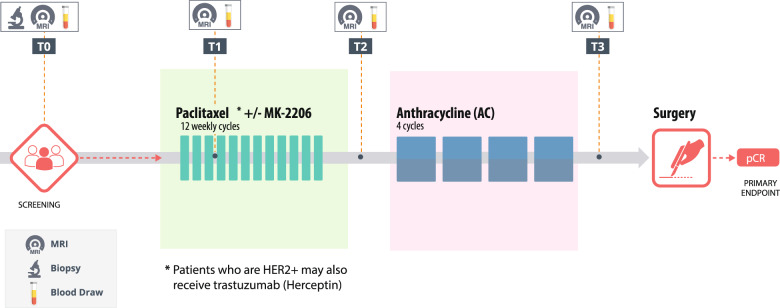
Study schema showing time points for collection of MRI-based functional tumor volume (FTV) and circulating tumor DNA (ctDNA) data during neoadjuvant chemotherapy (NAC). Patients received paclitaxel-based treatment followed by anthracycline-based chemotherapy. Data and biospecimens were collected at pretreatment (T0), 3 weeks after initiation of paclitaxel-based treatment (T1), between paclitaxel and anthracycline regimens (T2), and after NAC prior to surgery (T3). Pathologic complete response (pCR), the primary endpoint of this study, was assessed at surgical time point.

Data and biospecimens were collected at pretreatment (T0), 3 weeks after initiation of paclitaxel-based treatment (T1), between paclitaxel and anthracycline regimens (T2), and after NAC prior to surgery (T3) (Fig. 5). Hormone receptor (estrogen and progesterone) and HER2 status, and MammaPrint risk classification (MammaPrint high 1 and high 2) based on a 70-gene signature test were assessed at pretreatment.

ctDNA was detected using a personalized and a tumor-informed multiplex PCR next generation sequencing platform (Signatera^TM^ bespoke mPCR-NGS assay) as previously described^16^. Briefly, the test’s tumor-informed approach allowed for the detection of ctDNA by tracking up to 16 clonal variants (i.e., targets) in plasma. The patient-specific targets were based on the whole exome sequencing data of the untreated primary tumor. A sample was deemed ctDNA-positive when ≥2 targets were detected. ctDNA data were treated as dichotomous (ctDNA-positive or ctDNA-negative) or as continuous variables (mean tumor molecules per mL of plasma or MTM).

MRI data were acquired using a pre-specified imaging protocol and dedicated breast radiofrequency coil^10,11^. The image acquisition protocol included T2-weighted, diffusion-weighted, and dynamic contrast-enhanced (DCE) sequences performed bilaterally in the axial orientation. DCE-MRI was performed once pre-contrast and multiple times post-contrast using a 3D fat-suppressed T1-weighted protocol. Post-contrast imaging continued for at least 8 min following contrast agent injection. Gadolinium contrast agent was administered intravenously at a dose of 0.1 mmol/kg body weight, and at a rate of 2 mL/s, followed by a 20-mL saline flush. FTV was calculated by summing all voxels meeting a percentage enhancement threshold of 70% at approximately two and a half minutes post-contrast within a manually delineated box encompassing the lesion.

The biomarkers in this paper were evaluated and reported following REMARK guidelines^34^.

### Study design

The response endpoint for this study was pCR, defined as the complete eradication of invasive cancer in both the breast and regional lymph nodes, determined at the time of surgery. The survival endpoint was distant recurrence-free survival (DRFS), calculated from the date of patient consent for treatment to the date of clinical diagnosis of metastatic recurrence or death by any cause. Age at diagnosis, treatment, subtype, clinical T and N stage, MammaPrint score (as risk categories MammaPrint high 1 and MammaPrint high 2) and pCR were covariables in included in the Cox regression models. Patients were censored at the time of their last visit. Survival analysis was performed on follow-up data available as of February 20, 2019. The median follow-up time was 4.8 years (range: 0.5–6.3 years).

### Statistical analysis methods

Correlations between continuous ctDNA (MTM) and FTV (cubic centimeters) values were tested using Spearman’s rho (ρ). The median FTVs between ctDNA-positive and ctDNA-negative groups were compared using Wilcoxon rank sum test. The association between ctDNA and FTV temporal patterns in individual patients was assessed using Monte Carlo permutation test in the subset of patients who were ctDNA-positive at baseline. The analysis was performed as follows: a. Pearson correlation coefficients were calculated for serial FTV and the corresponding ctDNA concentrations (MTM/mL); b. Fisher z-transformation was performed on Pearson correlation coefficients in part (a) to normalize the distribution of the coefficients, thereby allowing more accurate calculation of the mean^35^ (regarded as observed mean); c. Analysis in parts (a) and (b) was repeated 1000 times with randomly permuted sample labels: the Pearson correlation coefficients between randomly paired samples were calculated and Fisher z-transformed. An empirical *p* value was calculated by comparing the actual observed data in (b) vs. theoretical data, i.e., the distribution of 1000 means generated by the random permutations in (c). A two-sided statistical was used to evaluate whether FTV and ctDNA were correlated at the individual patient level.

We have previously established subtype specific FTV-based prediction models trained using MRI data from 990 I-SPY 2 patients^12^. In this study, multivariable logistic regression analysis was performed to assess the additive value of ctDNA to pCR prediction models based on FTV alone. The 84 patients in our present study were part of this 990-patient cohort. To avoid overfitting, we re-developed our FTV models using data from the 906 patients who were not in our present study. These re-trained models were then applied to generate probabilities of achieving pCR for each patient in the 84 patients of this study at each time point: T1, T2, and T3, referred hereafter as FTV-based predictors (and used as continuous variables). To build FTV + ctDNA integrated models, continuous ctDNA variables based on pretreatment time point value only or in combination with changes between pretreatment and T1, T2, or T3 were added to the model. 10-fold cross-validation was used to evaluate AUC among all combinations of integrated models (FTV + ctDNA). The final ctDNA and FTV combined model at each time point included only ctDNA variables that resulted in the highest AUC. The ctDNA-FTV combined model was dichotomized into high vs. low probability of pCR using distance to the top-left corner of the receiver operating characteristics (ROC) curve to define an optimal threshold. The predictive performance of this classifier (positive predictive value, negative predictive value, and accuracy) was compared to that of the FTV-only model, also dichotomized using the top-left criterion.

We performed univariable and multivariable Cox regression analysis to determine the prognostic impact of FTV ctDNA, adjusted for the effects of pCR and subtype. We chose pCR and subtype as covariates based on recent findings in the I-SPY 2 TRIAL showing a strong prognostic impact of these two variables on survival in neoadjuvant-treated patients^7^.

The proportional hazards assumption was tested for each variable in a Cox regression model fit using scaled Schoenfeld residuals^36^ as implemented in the function cox.zph() in the survival package in R. The function included a global test for the whole model. Analysis showed that the test for each of the variable and the global test were not statistically significant, indicating that the proportional hazards assumption was met (Supplementary Table 4).

To assess whether FTV and ctDNA provided additional prognostic information to pCR and subtype, we built multivariable models that included these two variables with or without FTV or ctDNA variables. We then compared the models using the Akaike Information Criterion (AIC) scores. Lower AIC scores indicate a better fit to the data^37^.

Spearman’s and Wilcoxon rank sum tests, logistic and Cox regression analysis were implemented in R version 3.4.1 (R Foundation for Statistical Computing, Vienna, Austria).

## Supplementary information

Supplementary Table 1-5

## Data Availability

The data generated and analyzed during this study are described in the following data record: 10.6084/m9.figshare.14075876^38^. Clinicopathologic, ctDNA, and FTV data that support the findings of this study are available in Supplementary Table 5, which is available in the Supplementary Files of the article. Clinicopathologic and response data have also been deposited in NCBI’s Gene Expression Omnibus and are accessible through GEO SuperSeries accession number https://identifiers.org/geo:GSE150576^39^.The full MRI data will be deposited in The Cancer Imaging Archive (TCIA) and the accession ID is anticipated to be released in mid-2021. When the MRI data become available, the metadata record associated with the group’s previous article (10.6084/m9.figshare.12912191)^40^ will be updated to include the TCIA data DOI. Prior to release, MRI data queries can be directed to the corresponding authors (W.L. and N.H.).

## Code availability

R scripts are available upon request.
