# Supplementary material for: Circulating tumor DNA and magnetic resonance imaging to predict neoadjuvant chemotherapy response and recurrence risk
Source: NPJ Breast Cancer. 2021 Mar 25;7:32. doi: 10.1038/s41523-021-00239-3 (PMC7994408; doi:10.1038/s41523-021-00239-3)
Supplement: Supplementary file 1 — Supplementary Table 1-5 [file 41523_2021_239_MOESM1_ESM.pdf]

Supplementary Table 1. Optimized models for predicting pCR with FTV and FTV + ctDNA (continuous) as covariates. Area under the curve (AUC) was calculated with 10-fold cross validation for multivariate models. T1: 3 weeks after initiation of paclitaxel-based treatment; T2: between paclitaxel and anthracycline regimens; T3: after NAC prior to surgery.

Time point	Model	N (pCR rate)	AUC (95%CI)	P-value
T1	FTV only	53 (34%)	0.59 (0.43, 0.75)	0.25
	FTV + ctDNA		0.69 (0.60, 0.88)	
T2	FTV only	40 (40%)	0.78 (0.64, 0.93)	0.64
	FTV + ctDNA		0.75 (0.56, 0.90)	
T3	FTV only	36 (39%)	0.85 (0.72, 0.98)	0.4
	FTV + ctDNA		0.79 (0.64, 0.92)	

*FTV refers to the FTV-based predictor, which is the probability produced by the subtype-specific FTV model (See Methods). FTV + ctDNA refers to continuous ctDNA variables combined with the FTV-based predictor.

Supplementary Table 2. Univariable and multivariable Cox regression analysis to determine association between FTV, ctDNA, pCR, subtype and distant recurrence-free survival. T3 represents the time point after neoadjuvant chemotherapy prior to surgery. TN refers to triple negative breast cancer (HR-HER2-).

Variables	Univariable						Multivariable (n= 58, number of events = 8)			
	n	No. of events	Hazard ratio	lower 95% CI	upper 95% CI	Wald test p-value	Hazard ratio	lower 95% CI	upper 95% CI	LR p-value
FTV at T3 (continuous)	70	9	1.03	1.01	1.04	0.0005	1.03	1.01	1.1	0.0191
ctDNA at T3	60	8	11.50	2.87	46.14	0.0006	14.25	2.27	89.3	0.0046
Subtype: HR+HER2- vs. TN	75	10	0.61	0.14	2.54	0.4930	0.81	0.16	4.1	0.7990
HER2+ vs. TN			0.63	0.12	3.23	0.5770	0.68	0.03	14.6	0.8078
Response: pCR vs. no pCR	75	10	0.27	0.03	2.13	0.2140	1.61	0.11	24.2	0.7288

Supplementary Table 3. Assessment of quality and fit of models to the survival data using Akaike information criterion (AIC). Lower AIC indicates better fit. T3 represents the time point after neoadjuvant chemotherapy prior to surgery.

Models	AIC	LR p-value
pCR + subtype	87.17	0.4
pCR + subtype + FTV at T3	73.28	0.05
pCR + subtype + ctDNA at T3	60.18	0.02
pCR + subtype + FTV at T3 + ctDNA at T3	55.85	0.004

Abbreviations: pCR- pathologic complete response, FTV-functional tumor volume, ctDNA-circulating tumor DNA, LR- likelihood ratio test.

Supplementary Table 4. Testing proportional hazards assumption using scaled Schoenfeld residuals. FTV-functional tumor volume, ctDNA-circulating tumor DNA, pCR-pathologic complete response. T3 refers to the time point after neoadjuvant therapy prior to surgery.

Variable	Chi square	Degrees of freedom	p-value
FTV at T3	0.53	1	0.46
ctDNA at T3	0.31	1	0.58
Subtype	1.72	2	0.42
pCR	0.00	1	0.95
Global	3.63	9	0.6

Supplementary Table 5. ctDNA, FTV and clinicopathologic data.

Research ID	Mean Tumor Molecules per mL at T0	ctDNA status at T0	Mean Tumor Molecules per mL at T1	ctDNA status at T1	Mean Tumor Molecules per mL at T2	ctDNA status at T2	Mean Tumor Molecules per mL at T3	ctDNA status at T3	FTV at T0	FTV at T1	FTV at T2	FTV at T3	Arm	HR (0=neg,1=pos)	HER2 (0=neg,1=pos)	MammPrint (High 1=0, High 2=1)	pCR (0=No, 1=Yes)	Clinical T stage	Clinical N stage	Grade	DRFS time (days)	DRFS event (0=No, 1=Yes)
809037	0	-	0	-	0	-	0	-	5.9099496	14.358384	2.6472422	0.41772542	Paclitaxel	1	0	0	0	NA	NA	II	755	0
547405	10.3	+	2.3	+	0	-	0	-	NA	NA	2.5068469	NA	Paclitaxel + MK-	0	0	1	0	T3	N1	NA	470	0
753109	0	-	0	-	0	-	0	-	3.7610937	2.0834201	1.0734641	0.84765737	Paclitaxel + MK-	0	0	1	1	T2	N0	III	1625	0
220471	1.3	+	0.9	+	1.3	+	0	-	3.8131943	4.8715275	2.8717041	1.2701388	Paclitaxel + Tras	0	1	1	0	T2	N0	III	1988	0
604199	51.3	+	1.1	+	0	-	0.8	+	13.927629	7.1433105	1.5867445	2.0685831	Paclitaxel + MK-	1	0	0	0	NA	NA	II	1234	1
506623	10.4	+	0	-	NA	NA	NA	NA	41.005095	31.611181	6.4945903	NA	Paclitaxel + MK-	0	0	1	0	T4	N1	NA	1539	0
172168	6.8	+	0	-	0	-	0	-	7.3352051	5.9633684	1.0213651	0.049446617	Paclitaxel	0	0	1	0	T2	N0	NA	1833	0
518000	4.1	+	0.7	+	6.3	+	0	-	50.113133	11.284428	6.1255373	NA	Paclitaxel + MK-	0	0	1	0	NA	NA	NA	NA	NA
778527	13.6	+	2.8	+	0	-	0	-	2.7076388	1.9659546	NA	0.42022095	Paclitaxel + MK-	0	0	1	1	T2	NA	NA	1868	0
345907	3.3	+	NA	NA	NA	NA	NA	NA	5.6919178	NA	NA	NA	Paclitaxel + MK-	0	0	1	0	T2	N0	III	NA	NA
863161	1.6	+	0	-	0	-	0	-	15.788196	2.7395832	0.34027776	0.13472222	Paclitaxel + MK-	0	1	0	1	T2	N0	II	1596	0
999733	0	-	0	-	0	-	0	-	6.6553744	2.7756151	0.16438515	0.62696442	Paclitaxel + MK-	1	0	0	0	T2	N0	II	1702	0
651145	1.6	+	0	-	0	-	0	-	19.638192	3.9020298	0.12991189	0.58507082	Paclitaxel + MK-	1	0	0	0	T2	N1	II	1978	0
614434	0.3	+	0	-	0	-	0	-	4.0733761	1.0359375	0.44348631	0.005024873	Paclitaxel + MK-	1	1	0	1	T2	N0	NA	1749	0
294265	35.1	+	0.8	+	9.5	+	0	-	21.894942	12.42004	1.282244	0.078304978	Paclitaxel + MK-	0	0	1	1	T3	N0	NA	NA	NA
654064	0	-	0	-	0	-	0	-	25.036732	17.030047	6.5815611	7.7846196	Paclitaxel + MK-	1	0	0	0	T2	N1	NA	1716	0
422454	0	-	0	-	0	-	0	-	24.733216	21.789185	10.183594	3.6174317	Paclitaxel + MK-	1	0	0	0	T3	N1	III	1761	0
929391	7.7	+	NA	NA	NA	NA	NA	NA	3.2137668	NA	NA	NA	Paclitaxel	0	0	1	0	T2	N1	III	NA	NA
317402	0	-	0	-	NA	NA	NA	NA	2.7388281	0.66341718	1.0843064	0.47853042	Paclitaxel + MK-	1	0	0	0	T1	N0	II	1433	0
130892	5.8	+	0	-	0	-	0	-	32.655144	14.508393	0.0962771	0.011677446	Paclitaxel + MK-	1	0	1	1	NA	NA	III	NA	NA
732137	38.6	+	NA	NA	NA	NA	NA	NA	44.503033	95.577429	40.004626	30.566821	Paclitaxel + MK-	0	0	1	0	T3	N1	II	1433	0
332655	33.6	+	0	-	NA	NA	NA	NA	20.135742	NA	NA	NA	Paclitaxel + MK-	0	0	1	1	T2	N1	NA	1797	0
535415	1.9	+	0.4	+	0	-	0	-	8.8209375	6.8578216	0.24421875	0.00375	Paclitaxel + MK-	0	1	0	1	T2	N1	III	1911	0
342959	1.7	+	0	-	0	-	0	-	12.834833	4.7432716	0.19400139	0.043203735	Paclitaxel + MK-	0	0	0	1	T2	N0	II	1629	0
729732	0	-	0	-	0	-	0	-	3.6303387	0.85335291	0.43438587	2.2782662	Paclitaxel + MK-	1	0	0	0	T2	N1	NA	2006	0
635632	0.6	+	0	-	0	-	0	-	3.1974589	0.33694601	0.03704642	0.011466749	Paclitaxel + MK-	0	1	0	1	T2	N1	II	1721	0
319324	0	-	0	-	0	-	0	-	13.425706	4.6496004	0.42	1.1105606	Paclitaxel + MK-	1	1	0	0	T2	NA	II	1700	0
748611	90.5	+	1.5	+	0	-	0	-	14.73063	6.2347373	0.3757057	2.0258447	Paclitaxel	1	0	1	1	T2	N0	III	2018	0
601489	225.6	+	1.3	+	0	-	0	-	338.442	164.55615	44.157656	125.30705	Paclitaxel + MK-	0	1	1	0	T4	N2	III	881	1
180663	14.7	+	0	-	0	-	0	-	60.025794	11.455341	5.9579799	7.5559273	Paclitaxel + MK-	1	0	1	0	T3	N1	III	2149	0
293142	2.4	+	0	-	0	-	0	-	41.150452	47.172817	15.809301	24.139289	Paclitaxel	1	0	0	0	T3	NA	NA	1769	0
706639	0	-	0	-	1.3	+	0	-	5.8002341	0.1341897	1.7074483	0.6408715	Paclitaxel	0	0	1	0	T2	N0	III	1946	0
116603	1.9	+	0	-	0	-	0	-	112.71028	36.201958	6.2002718	8.1525072	Paclitaxel	0	0	1	0	T4	N3	III	221	1
938719	3.7	+	NA	NA	0	-	NA	NA	16.889317	11.267097	7.1010655	5.8968434	Paclitaxel	1	0	1	0	T2	N0	NA	1884	0
219545	27.3	+	8	+	NA	NA	13.6	+	32.195013	31.87764	9.6408061	5.5357269	Paclitaxel	0	0	1	0	T2	N0	NA	1603	0
639663	34.7	+	4.2	+	41.5	+	14.8	+	24.565971	NA	13.899902	7.5080566	Paclitaxel + MK-	0	0	1	0	T3	N0	III	1052	1
294739	0	-	0	-	0	-	0	-	8.9127686	4.2822479	4.0302246	0	Paclitaxel	1	0	1	0	T2	N2	NA	483	1
706169	925.6	+	1276.1	+	2312.7	+	2.2	+	179.69242	203.35251	264.96516	63.422997	Paclitaxel	0	0	1	0	T4	N2	NA	187	1
520258	244.4	+	58.8	+	NA	NA	0	-	19.845485	52.651039	61.098955	2.0531006	Paclitaxel	1	0	1	0	T3	N1	III	1881	0
402384	90.5	+	NA	NA	NA	NA	NA	NA	6.4729797	102.65405	86.234546	1.7926392	Paclitaxel + MK-	0	0	1	0	T3	N1	NA	NA	NA
771813	3.5	+	1.1	+	51.6	+	0	-	6.5852997	7.0793996	12.056849	1.2413249	Paclitaxel	0	0	1	0	T2	N0	NA	1273	0
856159	0	-	0	-	0	-	0	-	1.8536825	1.728295	0.07039301	0.026397378	Paclitaxel + Tras	1	1	0	0	T2	N1	III	1878	0
839274	7.7	+	0	-	0	-	NA	NA	78.263355	16.652734	1.1542118	0.29101562	Paclitaxel + MK-	1	1	1	0	T4	N1	II	1783	0
136536	1.8	+	0	-	0	-	0	-	3.1832998	3.1144498	1.0975499	0.028349999	Paclitaxel	0	0	1	1	T2	N0	NA	308	0
688955	1.6	+	0.7	+	NA	NA	0	-	6.1773927	3.4997975	2.0228786	0.11310719	Paclitaxel	1	0	1	0	T2	N0	II	1567	0
700549	0	-	0	-	0	-	0	-	20.61875	19.544531	12.867188	3.7921875	Paclitaxel + MK-	1	0	0	0	T2	N0	II	371	0
284593	0	-	0	-	NA	NA	0	-	24.756475	20.985427	31.711917	4.5054571	Paclitaxel + MK-	0	0	1	0	T2	N1	NA	2149	0
801641	7.2	+	0.5	+	0	-	0	-	20.326172	5.446875	0.090625	0.057187502	Paclitaxel + MK-	0	0	1	0	T2	N1	III	1811	0
414971	3.8	+	0	-	0	-	NA	NA	2.3874749	0.31994998	0.12352499	0.026324999	Paclitaxel + MK-	0	0	1	1	T2	N2	NA	1806	0
573605	1.7	+	0	-	0	-	0	-	6.0367188	5.7757133	0.003125	0	Paclitaxel + MK-	0	0	1	0	T2	N0	III	1826	0
317641	0	-	0	-	0	-	0	-	3.0914063	0.06015625	NA	NA	Paclitaxel + MK-	0	0	1	1	T2	N1	III	1925	0
712276	0	-	0	-	0	-	0	-	9.350625	1.9497002	0	0.014818568	Paclitaxel + MK-	1	0	0	1	T2	N0	NA	1099	0
263143	246.4	+	0	-	NA	NA	NA	NA	92.380298	24.920559	60.829917	0.69278154	Paclitaxel + MK-	0	1	1	0	T3	N1	III	1918	0
343898	5.4	+	10.5	+	0	-	0	-	57.587007	22.491693	5.1629822	5.8509094	Paclitaxel + MK-	0	0	0	0	T2	N1	III	1736	0
963303	0	-	0	-	0	-	0	-	6.7570313	6.78125	5.1976563	0.18359375	Paclitaxel	1	0	0	0	T2	N1	II	1925	0
637566	126.5	+	1.8	+	0	-	NA	NA	38.029525	14.970595	2.5095741	0.76880722	Paclitaxel + MK-	0	0	1	0	NA	NA	III	NA	NA
755914	6.3	+	0	-	NA	NA	0	-	6.0885971	1.3123535	0.50271606	0.001763916	Paclitaxel + MK-	0	0	1	1	NA	NA	III	1888	0
426106	162.3	+	4.4	+	11.6	+	53.2	+	80.334088	35.543123	10.375351	8.5579801	Paclitaxel + MK-	1	0	1	0	T3	N1	III	2031	0
179369	5.5	+	0	-	0	-	0	-	62.279442	10.598804	2.3154013	0.3862722	Paclitaxel + MK-	1	0	0	0	T3	N1	II	1665	0
375954	2.4	+	0	-	NA	NA	NA	NA	14.577106	1.3260223	NA	NA	Paclitaxel + MK-	0	1	1	0	T3	N0	III	NA	NA
803677	11	+	1.5	+	NA	NA	0	-	24.0075	7.8628125	0.99193845	0.98369976	Paclitaxel + MK-	0	0	1	0	T1	NA	NA	1095	0
698094	0	-	0	-	0	-	0	-	8.6372764	6.2784861	4.493638	4.2543727	Paclitaxel	1	0	0	0	T2	N1	II	2006	0
287961	0	-	0	-	0	-	NA	NA	9.6623437	8.0029687	3.69375	2.3835937	Paclitaxel + MK-	1	1	0	1	T3	N0	II	1598	0
520986	0	-	0	-	0	-	0	-	10.31089	5.5114476	0.24246267	0.18972155	Paclitaxel + MK-	1	0	0	1	T2	N0	III	1912	0
252748	8	+	1	+	0	-	NA	NA	44.199818	22.718455	4.4947266	2.8497044	Paclitaxel	0	0	1	1	T2	N0	III	1777	0
609642	2.5	+	0	-	0	-	0	-	0.77197269	0.0336914	0	0	Paclitaxel + MK-	0	1	1	1	T2	N0	III	1869	0
493519	0	-	0	-	0	-	0	-	16.980925	15.113651	2.6158856	0.96447488	Paclitaxel	1	0	0	0	T2	N1	II	1592	0
834418	2.8	+	3.4	+	0	-	0	-	6.6193413	5.0359698	3.9742188	1.0741733	Paclitaxel	1	0	0	0	T2	N0	II	1257	0
123660	1.1	+	0	-	0	-	0	-	15.054134	1.8675401	1.465454	0.72046173	Paclitaxel + Tras	0	1	0	0	T2	N1	NA	1499	0
824234	0.7	+	0	-	0	-	0	-	71.769301	32.160938	8.778125	2.940625	Paclitaxel + Tras	1	1	0	1	T2	N0	II	1835	0
413961	121.1	+	9.2	+	NA	NA	NA	NA	12.332132	25.098729	24.313803	1.6376002	Paclitaxel + MK-	0	0	1	0	T2	N0	III	1735	0
490023	0.7	+	0	-	0	-	0	-	23.380444	3.0187134	1.0154663	1.0174438	Paclitaxel + MK-	0	1	1	1	T3	N0	NA	1304	0
885871	0	-	0	-	0	-	0	-	17.324595	5.754726	3.2487907	2.2985949	Paclitaxel	0	0	1	0	T2	N0	III	1697	0
515565	301.2	+	77.8	+	NA	NA	NA	NA	26.438816	14.735847	1.3297396	3.888021	Paclitaxel	0	0	1	0	T2	N0	III	556	1
517870	0.4	+	1.2	+	0	-	0	-	3.4670157	0.8025	0.66140625	0.08203125	Paclitaxel	0	0	1	0	T2	N0	III	1912	0
752372	184.6	+	9.9	+	NA	NA	NA	NA	27.363868	32.076355	4.4412892	3.2170167	Paclitaxel	1	0	0	0	T3	N1	II	1764	0
680881	48.2	+	280.5	+	21.2	+	112.7	+	75.819304	53.603673	52.941831	36.232392	Paclitaxel + MK-	1	0	1	0	T2	N0	NA	365	1
162562	6.9	+	0	-	0	-	0	-	6.6260168	2.6426446	0.87059087	0.11202132	Paclitaxel + Tras	0	1	1	1	T1	N0	II	667	1
421917	17.3	+	7.6	+	NA	NA	NA	NA	14.142749	18.014495	NA	NA	Paclitaxel	0	0	1	0	T3	N1	NA	820	1
970839	0	-	0	-	0	-	0	-	4.8182547	1.4423234	0.93417388	0.33436679	Paclitaxel	1	0	1	0	T2	N0	III	1755	0
796558	1.3	+	0	-	0	-	0	-	9.4066589	11.424243	1.7624817	0.57843018	Paclitaxel	0	0	0	0	T2	N0	III	1897	0
384830	93	+	0	-	0	-	0	-	57.024316	13.338501	0.68719482	0.8637207	Paclitaxel + MK-	1	1	0	0	T3	N0	III	NA	NA
361701	0	-	0	-	0	-	0	-	2.0348419	1.5867634	0.79610061	0.67211773	Paclitaxel + MK-	1	0	0	0	T2	N1	NA	1892	0
960963	9.2	+	0.5	+	NA	NA	NA	NA	14.323761	4.2625284	2.4477152	2.6561541	Paclitaxel + MK-	1	1	0	0	T2	N0	NA	2315	0
